# Clinical Outcomes of Enamel Matrix Derivate Used in Surgical and Non-Surgical Treatment of Peri-Implantitis: A Systematic Review of Clinical Studies

**DOI:** 10.3390/medicina58121819

**Published:** 2022-12-10

**Authors:** Raluca Moldovan, Alexandru Mester, Andra Piciu, Simion Bran, Florin Onisor

**Affiliations:** 1 Faculty of Dental Medicine, University of Medicine and Pharmacy “Iuliu Hatieganu”, 400006 Cluj-Napoca, Romania; raluca.moldovan007@yahoo.com; 2 Department of Oral Health, University of Medicine and Pharmacy “Iuliu Hatieganu”, 400006 Cluj-Napoca, Romania; 3 Department of Medical Oncology, University of Medicine and Pharmacy “Iuliu Hatieganu”, 400006 Cluj-Napoca, Romania; andra.piciu@umfcluj.ro; 4 Department of Maxillofacial Surgery and Implantology, University of Medicine and Pharmacy “Iuliu Hatieganu”, 400012 Cluj-Napoca, Romania; dr_brans@umfcluj.ro (S.B.); florin.onisor@umfcluj.ro (F.O.)

**Keywords:** enamel matrix derivate, peri-implantitis, peri-implant disease, surgical treatment, non-surgical treatment

## Abstract

*Background and objectives*: The aim of this systematic review was to assess the available evidence of using enamel matrix derivate in the treatment of peri-implantitis. *Materials and methods*: Three electronic databases (*PubMed*, *Scopus*, and *Web of Science*) were searched until August 2022 to identify relevant articles. The inclusion criteria consisted in human clinical studies that reported the use of enamel matrix derivate (EMD) in surgical and non-surgical treatment of peri-implantitis. The risk of bias was assessed using Cochrane risk of bias tool for randomized clinical trials (RCTs) and for non-RCTs ROBINS-I tool. *Results*: Clinical studies included were published between 2012 and 2022 and consisted of two randomized clinical trials (RCTs) for non-surgical therapy and two RCTs, three prospective cohort studies, and one retrospective case series in surgical therapy. Due to the heterogeneity of patients’ characteristics and assessment of peri-implant therapy, statistical analysis could not be achieved. *Conclusions*: The use of EMD indicated a positive effect on both surgical and non-surgical therapy. However, the available literature is scarce, with low evidence in non-surgical approach and modest evidence in surgical approach using EMD. More RCTs with standardize protocols are necessary to evaluate the efficacy of using EMD in both therapies.

## 1. Introduction

Peri-implantitis is considered a major and growing problem in dentistry as the number of patients receiving dental implant restorations increases [1]. Using dental implants to support restorations has achieved very satisfactory results in restoring patient function and aesthetics [1,2]. Implant stability and survival require healthy peri-implant soft and hard tissue [3]. However, dental implants may lose supporting bone even with successful osseointegration [1,2]. Peri-implantitis is defined as a severe inflammatory reaction following dental implant treatment, leading to destruction of supporting tissue [4].

Peri-implant tissue is the tissue surrounding the osseointegrated dental implants and can be divided into two different entities: soft tissue (peri-implant mucositis) and hard tissue components, including bone [2,5,6]. Peri-implant mucositis is a reversible biofilm-related inflammatory disease of the peri-implant soft tissue without persistent marginal bone loss [6,7]. The clinical symptom of peri-implant mucositis is marked inflammation, manifested as erythema, bleeding on probing, or tenderness on probing without loss of supporting bone [6,7], whereas peri-implantitis is an inflammatory lesion of the mucosa that affects the supporting bone with loss of osseointegration [2,8]. Redness, swelling, suppuration, and increasing bleeding and pocket probing depths (PPD) ≥ 6 mm are often observed at implants diagnosed with peri-implantitis [7].

The etiology of peri-implantitis is multifactorial. Both peri-implant diseases are infectious in nature and are induced by bacteria from dental biofilms [1,2,8]. Recent evidence reinforces the role of risk factors in the pathogenesis of peri-implantitis [9]. Risk factors are identified as biologic and prosthetics/occlusal. Systemic disease, systemic medications, smoking status, history of periodontitis, lack of oral hygiene, and anatomic variations of bone structure are some of the biological risk factors [6,9,10]. Overall, prosthetic/occlusal risk factors such as prosthetics designed, occlusal dysfunction, and retained cement in the absence of inflammation are not thought to increase the risk of peri-implantitis, but these factors may modify disease progression if inflammation is present [1,6,10].

The most challenging task in the management of peri-implantitis is biofilm removal in order to achieve re-osseointegration and promote the reduction of peri-implant pockets [1]. Peri-implant mucositis therapy includes mechanical debridement in conjunction with adjunctive antimicrobials [1,2,6]. Hand instruments including titanium or carbon-fiber curettes and/or ultrasonic devices with various tips may be used to remove supra- and sub-mucosal calculus and biofilm deposits [7].

Once peri-implantitis has been diagnosed, treatment should be initiated without delay [7]. In cases of marginal bone loss ≤2 mm, non-surgical therapy may be successful in resolving peri-implantitis. In the majority of cases with advanced bone loss >6 mm, surgical treatment is needed. Limitations of non-surgical therapy of peri-implantitis lesions are related to the difficulty in accessing the implant surface and decontaminating the implant’s undercuts and grooves [1,7,8].

The principles of surgical treatment of peri-implantitis are to improve the cleanliness of the implant surface and to modify the anatomy of the peri-implant soft and hard tissue to achieve re-osseointegration [1,2]. Numerous surgical treatment options have been proposed and can be broadly categorized: non-augmentative (open flap debridement and resective therapy) and augmentative [1,2,8,11]. Open flap debridement (OFD) is aiming at gaining access to the implant surfaces to facilitate decontamination only when the peri-implant bone loss is shallow [11]. Multiple approaches have been proposed for implant surface decontamination during peri-implant surgery, such as mechanical (curettes, ultrasonic devices of air–powder abrasive systems), chemical, and laser treatments [1,2,11]. Resective therapy is indicated when horizontal bone loss with exposed implant threads is present. This therapy involves reducing/eliminating pathological peri-implant pockets, apical positioning of the mucosal flap, or recontouring of the bone with/without implantoplasty [1,2,11].

An augmentative approach for peri-implantitis aims at regeneration of the bone defect, achieving re-osseointegration, and limiting the recession of peri-implant soft tissue [1,2,11]. Treatment procedures may include the use of various bone grafts (autogenous bone, alloplastic, xenogeneic, or allogenic bone substitutes), guided tissue regeneration (GTR), or combinations of the aforementioned [1,2,11,12]. To achieve a successful regenerative approach, research has mentioned the use of enamel matrix derivatives (EMD) [12,13,14]. EMD has been successfully used for periodontal regeneration in intrabony defects [12]. The potential beneficial effects of EMD into peri-implant defects might be the ability to improve the osteoconductivity of bone grafts and antimicrobial effect and have positive effects on wound healing and tissue regeneration [12,13,14]. 

In regards to the last statement, the aim of our systematic review was to assess the available evidence of using enamel matrix derivate in surgical and non-surgical treatment of peri-implantitis.

## 2. Materials and Methods

### 2.1. Eligibility Criteria

This systematic review was conducted under the recommendations of Cochrane Handbook for Systematic Reviews of Interventions [15] and the PRISMA guidelines [16]. The focused question was based on PICO (Participants, Interventions, Comparison, Outcome) criteria: “In patients with peri-implantitis (P), what is the efficiency of enamel matrix derivate (I) used in surgical interventions compared with non-surgical interventions (C) in terms of clinical peri-implant tissue changes (O)?”. The PICO elements were as follows:

- Participants: patient diagnosed with peri-implantitis or peri-implant mucositis;
- Interventions: surgical treatment of peri-implantitis or peri-implant mucositis using enamel matrix derivate;
- Comparison: non-surgical treatment of peri-implantitis or peri-implant mucositis using enamel matrix derivate;
- Outcome: changes in periodontal parameters recorded: probing depth (PD), clinical attachment loss (CAL), bleeding on probing (BOP), and radiographic bone level (RBL);
- Study design: clinical trials; prospective, retrospective studies; cohort studies, case-control studies; and case series.

The following exclusion criteria were applied: in vitro animal studies; no clinical trials, cross-sectional, and cohort studies; systematic/narrative reviews, case reports, monographs, and letters to the editor; missing data regarding periodontal parameters or no reports on the use of erythritol; insufficient/missing/unpublished data; and articles published in other languages than English.

### 2.2. Search Process

The three electronic databases (*PubMed*, *Web of Science*, *Scopus*) search was performed by two reviewers (R.M. and A.M.) until August 2022 to identify relevant articles. The search strategy was applied as follows: (enamel matrix derivate OR enamel matrix protein) AND (peri-implant health OR peri-implantitis OR peri-implant mucositis OR peri-implant disease OR open flap debridement OR surgical treatment OR regenerative treatment OR reconstructive treatment OR guided tissue regeneration). In the first literature selection stage, titles and abstracts were screened for eligibility according to the inclusion criteria. In the second stage, full-text potentially eligible articles were examined. In case of any disagreements, a third reviewer (F.O.) intervened with an additional discussion. 

### 2.3. Data Extraction

The following data from the included studies were taken: first author, year of study, country, study type, characteristics of participants, type of intervention, periodontal parameters, follow-up, outcomes, and conclusions. 

### 2.4. Risk of Bias Assessment 

The risk of bias for the RCTs was quantified using The Cochrane Collaboration’s tool [17]. The risk of bias for the non-RCTs was quantified using ROBINS-I tool [18].

### 2.5. Statistical Analysis 

The main goal of our research was to obtain a meta-analysis; due to heterogeneity of patients’ characteristics and assessment of peri-implant therapy, statistical analysis could not be achieved. 

## 3. Results

### 3.1. Study Selection 

**Figure 1:**
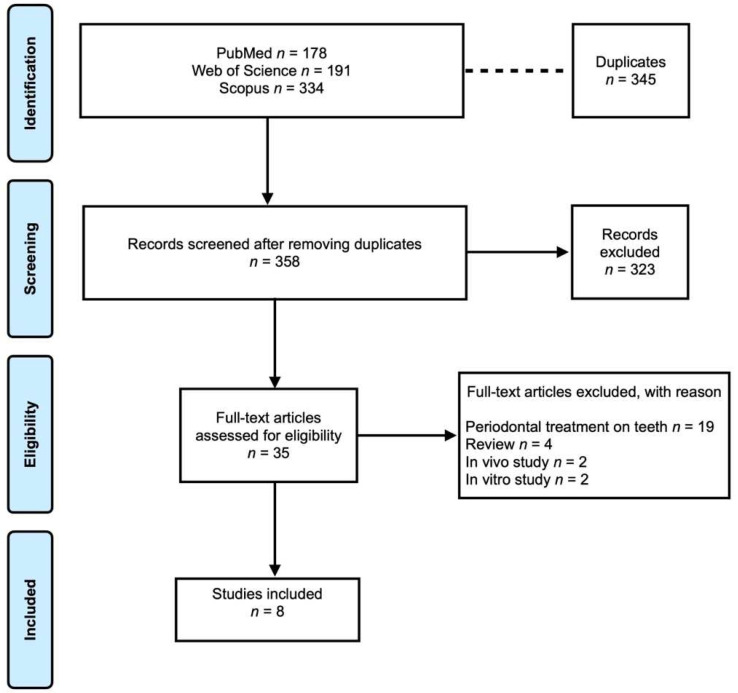
Prisma flowchart.

A total of 703 studies was retrieved from electronic databases. After removing the duplicates, a total of 358 studies was assessed through title and abstract. From the 35 articles that were full-text assessed, 8 studies met the inclusion criteria. The flow diagram according to PRISMA guidelines is shown in Figure 1. 

### 3.2. Study Characteristics

#### 3.2.1. Description of the Included Studies

Clinical studies included were published between 2012 and 2022 and were conducted in Iran, the USA, Sweden, Australia, and Switzerland. In regards to the study design, four RCTs, three prospective cohort studies, and one retrospective case series were included (Table 1).

#### 3.2.2. Characteristics of Studies Included

Two RCTs [19,20] evaluated the effects of EMD in non-surgical treatment of peri-implantitis. Surgical treatment of peri-implantitis using EMD was evaluated in three prospective cohort studies, one retrospective case series, and two RCTs.

##### Non-Surgical Treatment

In the RCT performed by Faramarzi and coworkers [19], EMD was compared to micro-spherical minocycline (MSM) as an adjunct to nonsurgical therapy for the treatment of peri-implant mucositis. A total of 64 patients (divided into three groups: control and two different test groups) were evaluated. A statistical significance was found for BOP reduction and PD reduction following EMD and MSM treatment. After 3 months, no significant changes in BOP and PD were found in the control group compared to the baseline. Alternatively, there was a significant decrease of BOP and PD in the experimental groups. In EMD group, BOP was reduced by 50%, and PD was reduced by 3 mm compared to the control group. The amount of decrease of BOP and PD in MSM group was 60% and 2mm, respectively.

The RCT from Kashefimehr and coworkers [20] used two different therapeutic protocols for the treatment of peri-implantitis that involved non-surgical mechanical debridement (MD) alone and MD with the application of EMD. This RCT included 41 patients with peri-implant mucositis, who were randomly divided into two groups (control and test group). The result showed that in the test group, PD and BOP were significantly reduced after 3 months, while no improvement was obtained in control group. The PD value after treatment with EMD was decreased to 3 mm, and the BOP was reduced by 50%.

Both RCTs included adults of 18 years of age or older, implants functional for at least one year with peri-implant mucositis and/or mild peri-implantitis defined as presence of BOP without soft tissue recession with or without minimal radiographic bone loss (≤2 mm), and a PD ≥ 4 mm [19,20]. If the subjects were smokers, substance/alcohol users, or undergoing radiotherapy for the head and neck area, or they had uncontrolled systemic disease, they were excluded. Further, if the PD ≥6 mm and if they had to use systemic antibiotics during the past 3 months, or they had any treatment intervention for peri-implant diseases during the past 3 months, patients were also excluded [19,20].

These two studies had a follow-up of 3 months. In the control groups, mechanical subgingival debridement was carried out using ultrasonic scaler instruments and glycine-based powder–air-polishing to remove subgingival biofilm [19,20]. In the EMD group, which represents the test group of both studies, after MD and isolation of the peri-implant area, according to Faramarzi et al. [19] and Kashefimehr et al. [20], 1 mg of EMD was placed subgingivally in the treated site. Despite that, in Kashefimehr’s study [20], EMD was applied 2 weeks after subgingival debridement; a 2-week healing interval should allow peri-implant soft tissue healing and reduce inflammation and bleeding to prevent EMD from flushing out of the sulcus. In the MSM group [19], after MD, 1 mg of minocycline hydrochloride microspheres were placed subgingivally in the treated area.

##### Surgical Treatment 

The RCT of Isehed et al. [21] from 2018 is the follow-up of the other RCT from the same group, Isehed et al., which was published 2016 [22], and one cohort study, namely Froum et al. from 2015 [23], is the continuity of the previous study from the same authors published in 2012 [24]. Another cohort study was published in 2018 by Mercado et al. [25]. In the end, our search included also a retrospective case series published by Pilenza et al. [26].

A total of 170 patients were included in the six studies; 4 patients dropped out, 154 patients received EMD treatment, and the rest were in the control group. Patients with confirmed peri-implantitis (PD ≥ 4 mm and BOP and at least one implant with angular peri-implant bone loss ≥3 mm) were included. Exclusion criteria were uncontrolled diabetes, heavy smoking (only Pilenza et al. [26] accepted smoking (<10 cigarettes per day)), and use of antibiotics or anti-inflammatory medication during the past 3 months [21,22,23,24,25,26]. 

In all studies, surgical treatment was performed after a full-mouth debridement, and all patients had to demonstrate adequate plaque control in order to continue with therapy. After the open flap, decontamination and debridement of the implant surface were performed using special graphite curettes, ultrasonic instruments, or titanium tips through different protocols. The use of tetracycline and air–powder abrasive with sodium bicarbonate powder was reported in two studies [23,24] and EDTA in another one [25]. In the study of Pilneza and coworkers [26], povidone iodine solution and airflow device were used for decontamination.

Follow-ups were preformed after 3, 6, and 12 months for PD and BOP [22,26]. In the three prospective cohort studies and one RCT, we selected data to evaluate the long-term (2 to 10 years) effects of the regenerative surgical treatment of peri-implantitis [21,23,24,25].

The two RCTs from Isehed and coworkers [21,22] aimed at comparing radiological and clinical effects of surgical treatment of peri-implantitis alone or in combination with EMD. According to Isehed et al. [22], the implant bone level increased from baseline to the 12 months follow-up with 0.9 mm in the EMD group, whereas it decreased by 0.1 mm in the control group. A stable radiographic bone level was obtained in 48% of the implants at the end of the 5-year follow-up. The mean bone-level gain was 1.4 mm [21]. In the Isehed et al. RCT [22], BOP decreased from 90% to 30% of the implants in either group, but it relapsed to 70% at the 12-month follow-up. Similar results regarding PD reduction were observed in the EMD group: 2.8 mm. Isehed et al. from 2018 found a BOP reduction of 44.4% in the EMD group and 60% in the non-EMD group at 5 years [21,22].

In Pilenza’s retrospective case series [26], after decontamination of the implant surface, hydroxyapatite was applied in combination with EMD and collagen membrane for GBR. Twenty implants were evaluated. The results showed a reduction in clinical and radiological parameters. The mean PD on the day of intervention was 4.9 mm; this value decreased after one year to a mean PD of 2.7 mm. At baseline, 18 (90.0%) out of 20 implants were diagnosed with BOP; at one year post surgery, only 4 (20%) implants exhibited BOP (*p* < 0.001). In terms of marginal bone loss, an average value of 6.0 mm at the mesial level and 4.9 mm at the distal level was found on the day of surgery. Measurements one year after surgery showed an average of 4.7 mm on the mesial side and 4.0 mm on the distal side of the implant, indicating an improvement in bone level [26].

The three cohort studies [23,24,25] used EMD with the addition of other regenerative materials such as PDGF + anorganic bovine/mineralized freeze-dried bone + SCTG/collagen matrix [23,24] or deproteinized bovine bone mineral with 10% collagen + doxycycline +/− CTG [25]. As a result, changes in mean bone level were observed; the mean pre-operative bone level was 3.80 mm, and the mean bone gain was 1.77 mm [23]. Before treatment, an average bone loss of 6.92 mm was observed by Mercardo’s study [25]; this was significantly reduced to 2.85 mm post treatment. BOP and PD were also significantly reduced; BOP was reduced from 168 to 15 when assessed preoperatively and compared to the final evaluation, representing a 91% reduction [23]. 

According to Mercardo and coworkers [25], 100% of treated implants had BOP at initial evaluation; this assessment dropped to 20% at both the 24- and 36-month follow-ups. The mean PD of the deepest peri-implant pocket of each implant at the initial visit was 8.9 mm; this mean PD reduced significantly to 3.55 mm by 12 months post treatment and was maintained at 3.50 mm at 24 and 36 months after the regenerative therapy [25]. Findings on mean PD reduction in the Froum’s study [23] showed a value of 5.10 mm with a range of 2 to 12 mm.

**Table 1:** Characteristics of the included studies.

Author. Year. Country	Study Type	Participant’s Characteristics	Type of Intervention	Periodontal Parameters	Follow-up	Outcomes
Non-surgical treatment						
Faramarzi. 2015. Iran [19]	Double-blind, three arm parallel RCT	MSM: *n* = 23 EMD: *n* = 20 Control: *n* = 21 Diagnosis: peri-implant mucositis (*n* = NA)	Mechanical debridement + MSM Mechanical debridement + EMD Mechanical debridement alone	PD BOP	2 weeks 3 months	PD reduction BOP reduction
Kashefimehr. 2017. Iran [20]	Double-blind RCT	Test: *n* = 20 Control: *n* = 21 Diagnosis: peri-implant mucositis (*n* = NA)	Test: mechanical debridement + EMD Control: mechanical debridement alone	PD BOP	3 months	PD reduction BOP reduction
Surgical treatment						
Froum. 2012. USA [24]	Prospective cohort	*n* = 38 Diagnosis: peri-implantitis (*n* = 51)	OFD with surface decontamination + EMD + PDGF + an organic bovine/mineralized freeze-dried bone + SCTG/collagen matrix	PD BOP RBL	3 to 7.5 years	PD reduction BOP reduction Bone-level gain
Froum. 2015. USA [23]	Prospective cohort	*n* = 100 Diagnosis: peri-implantitis (*n* = 170)	OFD with surface decontamination + EMD + PDGF + an organic bovine/mineralized freeze-dried bone + SCTG/collagen matrix	PD BOP RBL	2 to 10 years	PD reduction BOP reduction Bone level gain
Isehed. 2016. Sweden [22]	Double-blind RCT	Test: *n* = 15 Control: *n* = 14 Diagnosis: peri-implantitis (*n* = 29)	Test: OFD + EMD Control: OFD alone	PD BOP RBL	3, 6, 12 months	PD reduction BOP reduction Bone level gain
Isehed. 2018. Sweden [21]	RCT	Test: *n* = 13 Control: *n* = 12 Diagnosis: peri-implantitis (*n* = 25)	Test: OFD + EMD Control: OFD alone	BOP RBL	1 year 3 years 5 years	BOP reduction Bone level gain
Mercado. 2018. Australia [25]	Prospective cohort	*n* = 30 Diagnosis: peri-implantitis (*n* = 30)	OFD + EMD + deproteinized bovine bone mineral with 10% collagen + doxycycline +/− CTG	PD BOP RBL	1, 2, 3 years	PD reduction BOP reduction Bone level reduction
Pilenza. 2022. Switzerland [26]	Retrospective case series	*n* = 11 Diagnosis: peri-implantitis (*n* = 20)	OFD + EMD + hydroxyapatite + collagen membrane	PPD BOP RBL	1 year	PPD reduction

EMD, enamel matrix derivate; MSM, micro-spherical minocycline; BOP, bleeding on probing; CAL, clinical attachment level; OFD, open flap debridement; PD, pocket depth; PDGF, platelet-derived growth factor; PPD, probing pocket depth; SCTG, subepithelial connective tissue graft; RBL, radiographic bone level; RCT, randomized clinical trial.

### 3.3. Risk of Bias Assessment

None of the included RCTs, in both surgical and non-surgical therapy, achieved a low ROB (Table 2). In the RCTs from non-surgical therapy, Faramarzi et al. [19] achieved unclear risk, and Kashefimehr et al. [20] achieved high risk. In the RCTs from surgical therapy, both studies from Isehed et al. [21,22] achieved high risk. In non-RCT studies, Mercado et al. [25] and Pilenza et al. [26] achieved low risk; instead, studies from Froum et al. [23,24] achieved critical and moderate risk (Table 3). 

## 4. Discussion

Non-surgical and surgical treatment consensus have been published over the years in order to offer to clinicians a better perspective over the approach of peri-implantitis. In clinical practice, the definition of peri-implantitis is necessary in order to establish which treatment should be applied. In 2017, at the World Workshop, Berglundh and coworkers published a consensus paper of peri-implant disease and conditions [27]. Authors have stated that peri-implant health is characterized by the absence of erythema, BOP, swelling, and suppuration with no established PD threshold; further, peri-implant health may be compromised in implants with low bone support [27]. In peri-implant mucositis, clinical characteristics include BOP-positive with swelling and/or suppuration on probing, and PD may/may not be increased compared to previous examination; another feature is the absence of bone loss [27]. Besides the latter features of peri-implant mucositis, in peri-implantitis may be found increased PD and presence of bone loss; authors have stated that if there is no previous examination, peri-implantitis is defined as presence of BOP with suppuration with PD ≥6 mm and bone level ≥3 mm apical to the most coronal part of the intraosseous component of the implant [27]. As we have seen in our review, most of the studies included have used the clinical features of peri-implant mucositis and peri-implantitis.

Non-surgical treatment of peri-implantitis consists of mechanical debridement plus injection of EMD into the peri-implant sulcus. As seen in the Section 3, the adjunct use of EMD had positive outcomes, but with only two RCTs [19,20], a clear statement could not be achieved. Alberti and coworkers [13] found in their review that EMD was successfully used in peri-implantitis in 4 out of 11 papers included; however, for non-surgical treatment, the authors included the same studies as we included [19,20]. The systematic review from Bompard [28] found the same RCTs [19,20] as Alberti et al. [13] and our review in the non-surgical treatment of peri-implantitis. Both reviews [13,28] could not achieve statistical analysis due to heterogeneity of the data. 

Besides using EMD in non-surgical approach, the current literature has mentioned other alternative or adjunctive methods. Ramanauskaite and coworkers published a meta-analysis on the efficacy of non-surgical treatment of peri-implant mucositis and peri-implantitis [29]. Non-surgical treatments were alternative measures for biofilm removal and adjunctive methods (diode laser, antimicrobial photodynamic therapy, local antiseptics/antibiotics, systemic, probiotics) [29]. The authors’ results indicated that alternative measures for biofilm removal (weighted mean difference (WMD) −28.09%; *p* = 0.01) and systemic antibiotics (WMD −17.35%, *p* = 0.01) had great BOP reduction; the adjunctive use of local antiseptics (WMD −0.23 mm, *p* = 0.03) led to greater PD reduction, and changes in BOP were comparable (WMD −5.30%, *p* = 0.29) [29]. In the network meta-analysis of Hu and coworkers, authors compared different lasers in the non-surgical approach of peri-implantitis [30]. The authors’ results indicated that diode laser and conventional therapy had superior effect compared to conventional therapy alone in terms of PD reduction. Er:YAG and conventional therapy had greater efficiency compared to conventional therapy in terms of the plaque, clinical attachment level, and sulcus bleeding index. In the meta-analysis of Zhao and coworkers [31], the authors indicated that use of chlorhexidine did not improve PD (SMD 0.11; 95% CI: −0.16 to 0.38; *p* = 0.42, I2 = 0%) in peri-implant mucositis and also did not improve in peri-implantitis (MD = 1.57; 95% CI: −0.88 to 4.0; *p* = 0.21, I2 = 98%). The same authors mentioned that the efficacy of using chlorhexidine in reducing BOP in peri-implantitis is debatable [31]. 

Regarding the efficacy of using EMD in the surgical treatment of peri-implantitis, Alberti et al. found that EMD might improve bone–implant contact (BIC) although the long-term effects on bone stability and implant survival still need to be confirmed by more studies [13]. Bompard and coworkers mentioned in their systematic review that EMD combined with grafting materials improved the surgical treatment of peri-implantitis; in regards to the use of EMD alone, there is a lack of clinical studies to draw a clear conclusion [28]. Both reviews [13,28] could not achieve statistical analysis due to heterogeneity of the data. 

In the RCT of Isehed et al. [21], 31% of implants treated with EMD and 58% treated without EMD were lost due to reinfection. These percentages of lost implants seem very high compared to those found in the cohort studies included from Froum et al. [23,24]. The study of Isehed [21] documented failure of the technique in 2 out of 170 implants treated, denoting an overall survival rate of 98.8%. It can be observed that there is a disparity between RCTs and cohort studies in terms of implant survival [13,28].

To promote wound healing and tissue regeneration, Froum et al. [23,24] combined EMD with bone grafting and PDGF-BB. The cohort studies reported a mean increase in bone level (1.77 mm) and a greater reduction in PD (5.1 mm) and in BOP (91.10%) compared to the two RCTs in which a mean increase in marginal bone level (1.4 mm) and a smaller reduction in PD (2.8 mm) and BOP (44.4%) were observed [13,28]. This difference could be explained by the ability of PDGF-BB to improve bone filling in the treatment of periodontitis. Differences in clinical outcomes between cohort studies and RCTs using only EMD without any graft material may suggest that EMD alone has less ability to improve surgical treatment of peri-implantitis than when combined with other adjunctive materials [13,27].

Ramanauskaite and coworkers published a meta-analysis comparing the effects of adjuvant and alternative methods in the surgical treatment of peri-implantitis following a reconstructive versus a non-reconstructive approach [29]. Significantly higher PD reduction (−1.11 mm) was found at sites treated with adjunctive implantoplasty following surgical non-reconstructive peri-implantitis treatment. 

Regarding the rationale for systemic antibiotic administration after non-reconstructive peri-implantitis treatment, no differences in PD and BOP improvements were found between the test and control groups [29]. The same hypothesis regarding the administration of systemic antibiotics is supported by the work of Toledano-Osorio and coworkers [32]. The results indicated that in patients with peri-implantitis, the administration of systemic antibiotics reduced neither PD or BOP [32]. However, significant results could be obtained in terms of reducing clinical attachment loss, suppuration and peri-implant tissue recession, bone loss, and decrease of total bacterial count. Due to increasing resistance to antibiotics, authors recommended that administration should be done with caution [32]. 

Adjuvant reconstructive methods in conjunction with surgical treatment of peri-implantitis led to significantly higher radiographic bone defect fill/reduction (WMD 56.46%, *p* = 0.01; WMD −1.47 mm, *p* = 0.01), low PD (−0.51 mm, *p* = 0.01), and soft tissue recession (WMD −0.63 mm, *p* = 0.01), while changes in BOP (WMD −11.11%, *p* = 0.11) were not significant in the study from Ramanauskaite and coworkers [29]. The efficacy of reconstructive surgical therapy is consistent with the systematic review published by Tomasi and coworkers [33]. The results identified a greater improvement in marginal bone levels (WMD 1.7 mm) and in defect filling (WMD 57%), but found no difference for clinical measures of PD and BOP. In terms of overall outcome, therapy resulted in improved MBL (WMD 2.0 mm) and CAL (WMD 1.8 mm), recession (WMD 0.7 mm), reduced PD (WMD 2.8 mm), and reduced BOP (implants: RR = 0.4/Site: RR = 0.2) [33].

Our systematic review has several limitations. The first limitation would be the low number of RCTs and prospective studies included, which also had a low number of patients. Second would be the use of different diagnosis and treatment protocols, which may have represented a bias. Due to heterogeneity of the included data, a statistical analysis for a meta-analysis could not be achieved. Although the electronic literature search was limited to three electronic databases and was assessed until August 2022, several data may have been left out.

## 5. Conclusions

Our systematic review evaluated the efficiency of using EMD in surgical and non-surgical peri-implantitis. Our results indicated low evidence in non-surgical approaches and modest evidence in surgical approaches using EMD; however, a statistical analysis could not be achieved due to the heterogeneity of data. For these reasons, our results should be interpreted with caution. More RCTs with standardize protocols are necessary to evaluate the efficacy of using EMD in both therapies.

## Figures and Tables

**Table 2:** Cochrane RoB for RCTs.

Author	Adequate Sequence Generation	Allocation Concealment	Blinding of Participants and Personnel	Blinding of Outcome Assessment	Incomplete Outcome Data Addressed	Selective Outcome Reporting	Other Bias	Estimated Potential ROB
Faramarzi [19]	Low	Unclear	Low	Low	Unclear	Low	Low	Unclear
Kashefimehr [20]	Unclear	High	High	Unclear	Unclear	Low	Low	High
Isehed [21]	High	Low	Low	Low	Low	Low	Low	High
Isehed [22]	High	Low	Low	Low	Low	Low	Low	High

High, high risk of bias; Low, low risk of bias; Unclear, uncertain risk of bias.

**Table 3:** ROBINS-I for non-RCTs.

Author	Confounding	Selection of Participants	Classification of Interventions	Deviations from Intended Interventions	Missing Data	Measurements of Outcome	Selection of the Reported Result	Estimated Potential ROB
Froum [21]	Moderate	Critical	Moderate	Low	Low	Low	Moderate	Critical
Froum [22]	Moderate	Moderate	Moderate	Low	Low	Low	Moderate	Moderate
Mercado [25]	Low	Low	Low	Low	Low	Low	Low	Low
Pilenza [26]	Low	Low	Low	Low	Low	Low	Low	Low

Low, low ROB; Moderate, moderate ROB; Serious, serious ROB; Critical, critical ROB.

## Data Availability

Not applicable.
